# Markers of Bone Health and Impact of Whey Protein Supplementation in Army Initial Entry Training Soldiers: A Double-Blind Placebo-Controlled Study

**DOI:** 10.3390/nu12082225

**Published:** 2020-07-26

**Authors:** JoEllen M. Sefton, Kaitlin D. Lyons, Darren T. Beck, Cody T. Haun, Matthew A. Romero, Petey W. Mumford, Paul A. Roberson, Kaelin C. Young, Michael D. Roberts, Jeremy S. McAdam

**Affiliations:** 1 Warrior Research Center, School of Kinesiology, 301 Wire Road, Auburn University, Auburn, AL 36849-5323, USA; kdm0031@auburn.edu; 2 Molecular and Applied Sciences Laboratory, School of Kinesiology, 301 Wire Road, Auburn University, Auburn, AL 36849-5323, USA; dbeck@auburn.vcom.edu (D.T.B.); haunct@goldmail.etsu.edu (C.T.H.); mzr0049@auburn.edu (M.A.R.); pwm0009@auburn.edu (P.W.M.); par0021@auburn.edu (P.A.R.); kyoung@auburn.vcom.edu (K.C.Y.); mdr0024@auburn.edu (M.D.R.); 3 Department of Cell Biology and Physiology, Edward Via College of Osteopathic Medicine (Auburn Campus), Auburn, AL 36849-5323, USA; 4 Fitomics, LLC., Birmingham, AL 35294, USA; 5 Department of Cell, Developmental, & Integrative Biology, Center for Exercise Medicine, University of Alabama at Birmingham, Birmingham, AL 35294, USA; jmcadam@uab.edu

**Keywords:** biomarker, bone injury, injury prevention, military training, musculoskeletal injury, stress fracture

## Abstract

Training civilians to be soldiers is a challenging task often resulting in musculoskeletal injuries, especially bone stress injuries. This study evaluated bone health biomarkers (P1NP/CTX) and whey protein or carbohydrate supplementations before and after Army initial entry training (IET). Ninety male IET soldiers participated in this placebo-controlled, double-blind study assessing carbohydrate and whey protein supplementations. Age and fat mass predicted bone formation when controlling for ethnicity, explaining 44% (*p* < 0.01) of bone formation variations. Age was the only significant predictor of bone resorption (*p* = 0.02) when controlling for run, fat, and ethnicity, and these factors together explained 32% of the variance in bone resorption during week one (*p* < 0.01). Vitamin D increased across training (*p* < 0.01). There was no group by time interaction for supplementation and bone formation (*p* = 0.75), resorption (*p* = 0.73), Vitamin D (*p* = 0.36), or calcium (*p* = 0.64), indicating no influence of a supplementation on bone biomarkers across training. Age, fitness, fat mass, and ethnicity were important predictors of bone metabolism. The bone resorption/formation ratio suggests IET soldiers are at risk of stress injuries. Male IET soldiers are mildly to moderately deficient in vitamin D and slightly deficient in calcium throughout training. Whey protein or carbohydrate supplementations did not affect the markers of bone metabolism.

## 1. Introduction

Training civilians to be soldiers is a challenging task that must be successfully completed in a limited amount of time (9–16 weeks). Preparing new soldiers physically to withstand the rigors of the profession without injuring them is one of the most challenging parts of this process. Musculoskeletal injuries (MSI) are a primary cause of lost training time, recycle (repeating training), and attrition in US Army initial entry training (IET). MSIs are a universal problem in this population, with all military branches [1,2,3,4,5,6,7,8,9,10] and multiple nations [11,12,13,14,15] combating the same issues. The low level of recruit fitness is a serious and worsening problem [16] that mirrors the poor fitness and nutritional status of the population as a whole. [17,18,19] MSI impact the retention of new and experienced service members and overall force readiness. Improved knowledge regarding how a soldier’s physiology interacts with all the factors involved in military training may contribute to the design and implement of effective MSI reduction strategies.

Stress injuries (stress fractures and stress reactions) are a potentially serious MSI that plagues IET. A 2012 study found incidences of stress fractures in IET training to be 19.3 and 79.9/1000 recruits for men and women, respectively [20]. Stress injuries force IET soldiers to miss training for extended periods of time (averaging 85–116 days depending on the location of the fracture). [14] Stress injuries can occur when there is an imbalance in the bone remodeling response to mechanical stress (such as physical training). This imbalance is characterized by an increase in the resorption of microdamaged bone (by osteoclasts) and the insufficient reformation of the resorbed bone by osteoblasts. Research indicates that bone turnover may be increased in military personnel [21,22,23]. The biomarker serum procollagen type 1N propeptide (s-P1NP) has been used to assess bone formation and as a predictor of future bone fractures [24]. The serum C-terminal crosslinking telopeptide of type 1 collagen (s-CTX) can be used to assess bone resorption [24,25]. The P1NP/CTX ratio provides information on the amount of bone formation to bone resorption as an indicator of metabolism and health [26,27,28]. Vitamin D and calcium are also indicators of bone health [22,29]. One study found female soldiers in IET experienced dynamic changes in vitamin D status and increased bone turnover, while intakes of calcium and vitamin D were inadequate [22]. Studies have also found supplementations with calcium and vitamin D decrease the incidence of stress fractures in military personnel [30], maintains parathyroid hormone levels, and improves bone density in IET [31].

Several studies have indicated that IET soldiers do not consume the necessary nutrients to sustain the unfamiliar and often intense military training encountered during IET [32,33,34]. Supplementations with whey protein (WP) have been found to improve muscle mass, strength [35], bone metabolism [36,37], and performance [38], while reducing MSI [33]. However, to date, no studies have assessed biomarkers for bone health and WP supplementations upon entry and across IET soldiers. Thus, the goal of this study was to evaluate bone health biomarkers before and after IET training and the impact of WP versus carbohydrate-only supplementations on these markers of bone health.

## 2. Materials and Methods

### 2.1. Study Design and Population

**Figure 1:**
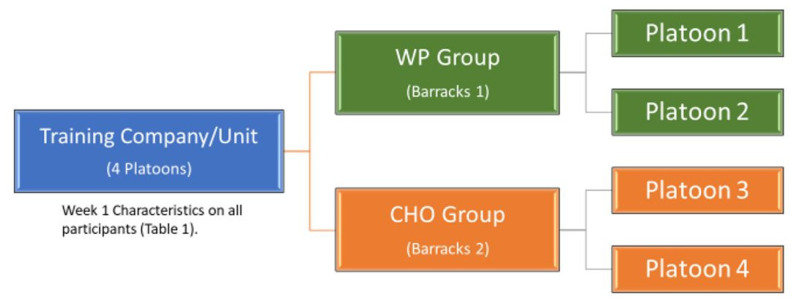
Grouping of the participants. WP: whey protein and CHO: carbohydrate.

This nine-week study was governed by a placebo-controlled, double-blind, 2 × 2 (group × time) factorial-repeated measures design. This study protocol was approved by the Auburn University IRB, Auburn AL, protocol #15-502 MR 1512 originally approved on 9 December 2015. A total of 90 healthy, male IET soldiers consented to participate. Participants were between 19 and 35 years old and participating in Army IET between March and April 2018 at Fort Benning, GA, USA, with no apparent disease or MSI, free from allergies to milk or whey proteins, and not supplementing or have supplemented within the past 3 months. Four participants were removed from the analysis due to prior supplementation (discovered during the study), and four participants were removed due to a lack of adherence to consuming supplementations. Five participants missed the final data collections and were removed. This study was approved by the Auburn University Institutional Review Board, Auburn AL, and the Army Institutional Review Board and Director, Research & Analysis Directorate Army Center, Training and Doctrine Command, Fort Eustis, VA, USA. Potential participants were briefed by the study team on the study purpose and procedures. Those wishing to participate read and signed the informed consent documents. The study protocols were completed as previously described [32,33]. The Army training unit (company) had four platoons; each platoon trained together and were housed together. We chose to use the two platoons housed in barracks one as the WP group and the two platoons housed in barracks two as the carbohydrate (CHO) group, rather than randomizing the groups by individual soldiers. This helped prevent the sharing and trading of the supplements, reduced the time impact on the Army cadre that would be distributing the supplements, and reduced the opportunity for mix-ups (Figure 1). The Army cadre only had access to the appropriate supplements for their platoon. Everything was also color-coded, which allowed for a quick visual check that everyone had the correct supplement. Everyone participating in the study and the research team was blinded to which supplement contained the WP or the CHO until after the study results were analyzed.

Assessing all the participants, we found no significant differences in characteristics between the participants making up the two groups (Table 1). This allowed us to assess the differences between the two groups at the end of the study.

### 2.2. Intervention

Servings were provided in single-serving packets that were blinded for contents to the study staff, drill sergeants, and IET soldiers. Soldiers in 2 platoons consumed one serving per day of whey protein (WP) in liquid-shake form; soldiers in the remaining 2 platoons consumed one serving per day of carbohydrate (CHO) in liquid-shake form. Supplements were manufactured at JW Nutritional, LLC (Allen, TX, USA), a United States Food and Drug Administration Current Good Manufacturing Practice compliant facility independently audited and prequalified by Obvium*Q, LLC (Phoenix, AZ, USA), a GMP regulatory compliance firm. Personnel at JW Nutritional, LLC and Lockwood, LLC (Draper, UT, USA) formulated supplements to match for taste and maintained blinding of the groups. Each supplement was assigned a randomly generated item number. The research team and participants were blinded to the contents of the packets until data collection was completed. Manufacturing batch records for the production of each of the supplements were reviewed by a trained, independent expert in dietary supplement quality control, taste, and assurance (C.M.L.). The nutritional profile and amino acid contents of both supplements were third-party tested by Covance Laboratories, Inc. (Madison, WI, USA) to verify the identity, purity, potency, and composition of the packets. One WP serving provided 293 total kcals, consisting of 38.6 g of protein (Power Crunch^®^ ProtoWhey^®^ (BioNutritional Research Group, Irvine, CA, USA) as agglomerated, partially hydrolyzed (12.5% degree of hydrolysis) 80% whey protein concentrate (Hilmar^®^ 8360; Hilmar Ingredients, Hilmar, CA, USA)), 19.0 g of carbohydrates, 7.5 g of fat, 20.1 g and 9.5 g of essential and branched chain amino acids, and 510 mg of calcium per 100 g of protein. One CHO serving provided 291 total kcals, 0.5 g of protein, 63.4 g of carbohydrates, 3.9 g of fat, and 0.1 g and 0.0 g of essential and branched chain amino acids. A member of the research team delivered supplements weekly and assessed the supplements and distribution for compliance. Participants also self-reported any missed servings to the drill sergeant and a researcher [32]. IET soldiers were introduced to training and completed their first physical fitness assessment on days one and two. They consumed their supplements mixed with water prior to bed, beginning on day 3 of training and continuing through week 8 of training.

### 2.3. Outcome Measures

**Figure 2:**
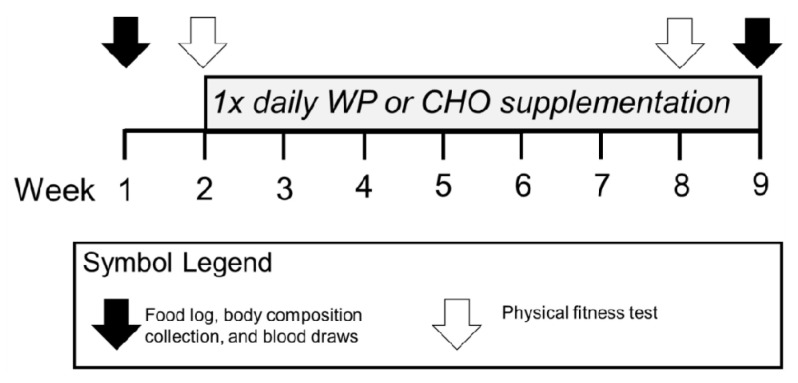
Timeline for outcome measures and interventions for this nine-week study.

Following consent, we collected demographic, phenotypic, and biological data. Phenotypic data consisted of body measures [height, weight, fat mass (FM)], and fat-free mass (FFM)) and physical performances [push-ups (PU), sit-ups (SU), and run time]. Biological data consisted of a blood analysis of P1NP, CTX, vitamin D, and calcium. All body measures and biological data were collected in the morning, after an overnight fast, and prior to breakfast and morning physical training. Figure 2 summarizes the nine-week study timeline.

### 2.4. Body Measures

Hydration can influence body measures. To control for this, we assessed the hydration status via urine-specific gravity (USG) prior to all body measures/blood draws. Participants with USG below 1.03 were considered adequately hydrated. Those above 1.03 were given water to drink and not allowed to proceed with testing until urine-specific gravity was below 1.03.

### 2.5. Height and Weight

Height (centimeters) and weight (kilograms) were assessed with IET soldiers wearing only army-issued physical training shorts, socks, and shirts using a Health-O-Meter professional scale (Model 500KL, Sunbeam products INC., Boca Raton, FL, USA).

### 2.6. Body Composition

Body composition (FM and FFM) was evaluated and used as a predictor variable in our regression models. FM and FFM were measured using a four-lead bioelectrical impedance device (ImpediMed DF50, Carslbad, CA USA). Participants were asked to lay supine for five minutes to allow for equilibration of fluids in their body compartments. Then, the left wrist and ankle were shaved, and electrodes were placed on the left wrist (midline, top electrode, even with the ulnar styloid process, and bottom electrode, 5-cm distal), and two other electrodes were placed on the midline of the left leg (superior electrode, even with the lateral malleolus, and distal electrode, 5-cm inferior). FFM and FM were calculated from the raw output using the formulas below:

FFM = (Height) 2/Resistance × 0.734 + 0.116 + Reactance × 0.096 + 1 × 0.878 − 4.03(1)

FM = Body Mass − FFM(2)

Height was input into the FFM calculations in centimeters, and body weight was in kilograms. Both FFM and FM are represented as mean ± standard deviation in kilograms.

### 2.7. Physiological Biomarkers

Fasted blood draws were collected in 10-mL serum separator vacutainer tubes (BD Vacutainer, Franklin Lakes, NJ, USA) from the antecubital vein by a trained member of the research team. Samples were placed on ice in a cooler (Yeti Coolers LLC, Austin, TX, USA) for transportation to the laboratory for processing. The blood samples were centrifuged at 3500× *g* for 10 min at room temperature. Serum was extracted and stored at −80 °C (Kendra Laboratory Products, Asheville, NC, USA) until analysis.

We evaluated four biomarkers related to bone health: C-terminal crosslinks of type 1 collagen (CTX; bone resorption), procollagen I intact N-terminal (P1NP; bone formation), vitamin D, and calcium. CTX and vitamin D were analyzed from serum using Enzyme-Linked Immunosorbent Assays (ELISA; USA immunodiagnostic Systems Inc., Gaithersburg, MD, USA). All samples were run in duplicate for ELISA kits, and each participant’s pre- and post-samples were run on the same plate. All optical densities were within the detectable range of the assay and read on a multispectral spectrophotometer (BioTek Eon, Winooski, VT, USA) at the manufacturer’s recommended wavelengths. Serum concentrations of each optical density were calculated using a 4-parameter logistic regression, as per the manufacturer’s recommendations.

Procollagen I intact N-terminal (P1NP) evaluated using chemiluminescence. Plates were read at respective wavelengths using a multispectral spectrophotometer (BioTek Eon, Winooski, VT, USA). Serum samples were analyzed for calcium by the Clinical Laboratory Improvement Amendmentscertified laboratory at East Alabama Medical Center in Auburn-Opelika, AL, USA.

### 2.8. Statistical Analysis

Our first aim was to characterize the parameters of bone health upon entry to IET and determine if there were any demographic or phenotypes that are indicative of bone health in IET soldiers.

First, we conducted bivariate testing to identify potential target variables to include as predictors in our regression models. We identified bivariate relationships using the chi-square analysis on categorical predictors (ethnicity) and Pearson’s correlation continuous predictors (age, body mass, fat-free mass, fat mass, push-ups, and run performance) with markers related to bone health (vitamin D, calcium, P1NP, and CTX). From our correlation matrix, potential relationships were identified between age, fat mass, and run performance with P1NP and CTX. We also analyzed correlations between the performance, body composition, and all the serum variables. There was no significant correlation between any of the biomarkers, with interleukin (IL)-6 being the closest, with a correlation of −0.28 and a *p*-value of 0.071.There were significant correlations between the body weight and fat-free mass with our predictor variables; however, we did not include these variables in our model. We chose to exclude those variables because (1) they were highly correlated with the fat mass and would inflate the variance in our model, and (2) because we used a 4-lead bioelectrical impediance analysis(BIA) device, the FFM is calculated from the body weight and estimated FM; therefore, this would explain the high correlation between the variables and inflate the variance in our model and provide no further benefit to the predictions. Therefore, we continued with our analysis using only the previously mentioned variables (ethnicity, run performance, FM, and age).

We conducted normality testing on the residuals of both models and tests for constant variance, as well as inspected the models for influential observations. Reviewing the influential observations revealed some values that seemed to exert leverage and influence on the models. However, an examination of the residual plots and scatterplots of the predictor variables plotted against the outcome variables determined that the values were within reason physiologically and followed a similar trend as the remaining data. Therefore, the values were not removed from the analysis.

Our second aim was to evaluate the influence of training and protein supplementation on these markers of bone health (P1NP, CTX, vitamin D, and calcium). To evaluate this, we used a 2 × 2 factorial ANOVA with the supplementation group (WP versus CHO) and time (pre versus post) as our independent factors. We first evaluated the interaction, and then, if no interaction existed, the main effects of time and group. Where significant group-by-time interactions were detected, we conducted Tukey’s honest significant difference tests to determine which pairs of means were different between each level of our independent variables of interest. Prior to conducting our ANOVA, we tested the assumption error normality and homogeneity of variance. We evaluated the error normality visually, using qqplots and residual plots and then statistically using a Shapiro-Wilks test. We evaluated the homogeneity of variance assumption visually using residual plots and statistically using the Levene’s test for homogeneity of variance. To assess the clinical relevance of our intervention on our outcome variables, we calculated Cohen’s D effect sizes for each group across the training. The effect size calculations are listed below.

Effect Size = Mean_(post)_ − Mean_(pre)_/SD_pooled_ (3)

SD_pooled_ = Square root ((SD_(pre)_^2^ + SD_(post)_^2^)/2) (4)

## 3. Results

### 3.1. Baseline Analysis

A total of 42 participants were included in the P1NP analysis, a biomarker for bone formation. A forward and reverse stepwise regression model revealed that age, fat mass, and ethnicity contributed to the most accurate prediction of bone formation. The coefficients for each predictor are listed in Table 2. The overall stepwise regression model was significant (F _(3,38)_ = 11.67, *p* = 0.00). Together, these variables explained 44% (adj. R^2^ = 0.44, *p* < 0.01) of the variations in baseline bone formations in IET soldiers. Age (*t* = −3.08, *p* < 0.01) and fat mass (*t* = −3.92, *p* < 0.01) were significant predictors of bone formations when controlling for each of the other variables in the model. For every one-unit increase in age, we found a decrease of 4.39 units in bone formation, when controlling for fat and ethnicity. There was an inverse relationship for fat mass; for every one-unit increase in fat mass, there was a decrease in bone formation of 3.06 units. Ethnicity was not found to be a significant predictor of bone formation (*p* = 0.10) when controlling for age and fat mass. In general, age (when controlling for fat and ethnicity) and fat mass (when controlling for age and ethnicity) are inversely related bone formations.

A total of 59 participants were included in the CTX analysis, a biomarker of bone resorption. A forward and reverse stepwise regression model was completed; run performance, age, fat mass, and ethnicity were included in the final model (Table 2). The overall stepwise regression model was significant (F _(4,54)_ = 6.32, *p* < 0.01) and accounted for 32% of the variance in bone resorptions in IET soldiers at baseline (adj. R^2^ = 0.32, *p* < 0.01). Age was the only significant predictor of bone resorption (*t* = −2.35, *p* = 0.02) when controlling for run, fat, and ethnicity. For every one-unit increase in age, there was a related decrease in bone resorptions by 26.63 units. Although these variables had low *p*-values, none were significant predictors (run (*t* = −1.56, *p* = 0.12), fat (*t* = −1.96, *p* = 0.06), and ethnicity (*t* = −1.60, *p* = 0.11)) when controlling for all other respective predictor variables. Overall, age was significantly associated with bone resorptions when accounting for the relationships between bone resorption and fat mass and run performance and ethnicity.

### 3.2. Effects of Supplements

The overarching hypothesis was that WP would have a beneficial effect on the biomarkers of bone health. The number of participants analyzed for each biomarker were 42 for P1NP, 48 for CTX, and 40 for calcium and vitamin D; sample size differences were a result of limited serum availability for some participants.

The effect of training and supplementations on P1NP was evaluated in 42 participants (WP = 20 and CHO = 22). There was no significant group-by-time interaction for bone formation (P1NP) (F = 0.1, *p* = 0.75), main effect of time (F = 1.73, *p* = 0.2), or group (F = 0.18, *p* = 0.68).

The effect of training and supplementations on CTX was assessed in 48 participants (WP = 23 and CHO = 25). There was no significant group-by-time interaction for CTX (F = 0.12, *p* = 0.73), main effect of time (F = 0.01, *p* = 0.94), or group (F = 0, *p* = 0.95). We evaluated the effect of training and supplementations on bone turnover by the ratio of bone formation to bone resorption (P1NP/CTX ratio). This metric provides an index of the amount of bone turnover instead of formation or resorption alone. A total of 42 participants (WP = 20 and CHO = 22) were sampled. There was no significant group-by-time interaction for the P1NP/CTX ratio (F = 0.4, *p* = 0.53), main effect of time (F = 1.81, *p* = 0.19), or group (F = 0.59, *p* = 0.45).

**Figure 3:**
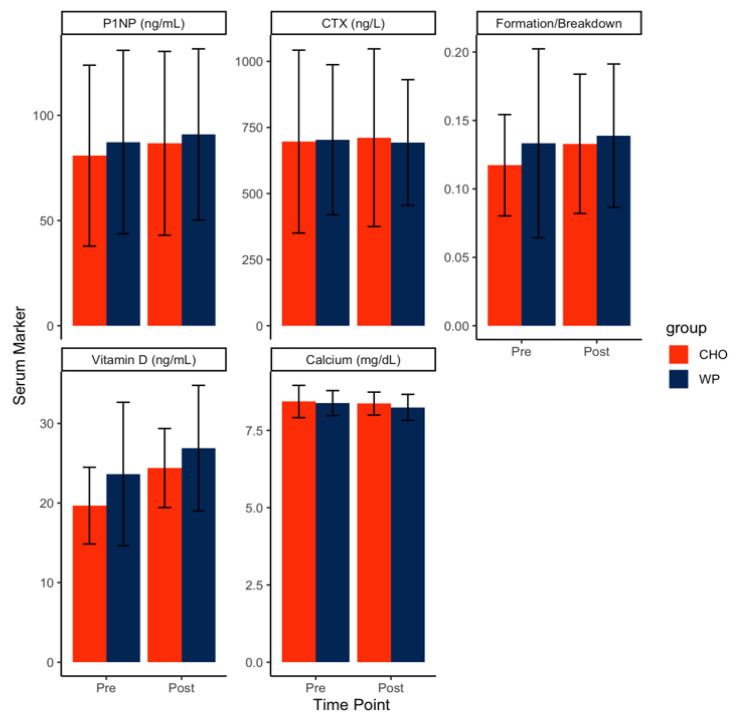
Physiological biomarkers of bone health across the initial entry training (IET). P1NP, procollagen type 1N propeptide and CTX, C-terminal crosslinking telopeptide of type 1 collagen.

We tested the effects of training and supplementation on vitamin D on a sample of 40 participants (WP = 18 and CHO = 22, Table 3). There was a significant main effect of time on vitamin D (F = 26.49, *p* < 0.01), indicating an increase in vitamin D across the training. There was no significant group-by-time interaction (F = 0.85, *p* = 0.36) or main effect of the group (F = 2.65, *p* = 0.11). The effects of training and supplementations on the serum calcium were assessed in 40 participants (WP = 26 and CHO = 24). There was no significant group-by-time interaction (F = 0.22, *p* = 0.64), main effect of time (F = 1.64, *p* = 0.21), or group (F = 0.54, *p* = 0.47) for the serum calcium (Figure 3).

## 4. Discussion

The purpose of this investigation was two-fold: (1) to characterize factors that may predict/be related to markers of bone health and (2) to evaluate the impacts of supplementations on markers of bone health. The major finding of this double-blind, placebo-controlled trial was male IET soldiers were mild to moderately deficient in vitamin D (19.9–26.9; healthy range is 50–75 nmol/L) [39] at the beginning of and throughout IET training. The slight increase in vitamin D levels across training was likely due to training outside in the sun in the Southeastern United States for nine weeks in the spring. Likewise, IET soldiers had a slight deficiency in calcium (8.2–8.4; normal range is 8.8–10.4 mmol/L), with no change in levels across the nine weeks of training. There was no impact of CHO or WP supplementations on either calcium or vitamin D levels.

Vitamin D plays an important role in maintaining bone health. Exposure to sunlight causes vitamin D_3_ to be produced in the skin. Differences in skin tone have been noted as a possible reason for differences in Vitamin D levels often found in different ethnicities [40]. Vitamin D_3_ is then metabolized to 1.25-dihydroxyvitamine D, which serves a vital role in maintaining serum calcium and phosphorus concentrations needed to maintain bone metabolism and skeletal mineralization, including increasing the calcium absorption from the gut and increasing the osteoclast reabsorption of bones. Plasma calcium levels are tightly controlled in a complicated feedback system involving primarily bones, which serves as calcium reservoirs, as well as the parathyroid gland, parathyroid hormone, vitamin D metabolites, and calcitonin [41]. Moderately low levels of calcium may promote bone resorption to free stored calcium and further inhibit the ability for soldiers to repair and remodel bones at the rate demanded during unaccustomed intense physical training.

Calcium serves an essential role during muscle contractions. Motoneurons release acetylcholine at the neuromuscular junction. Signals are then conveyed through the cell membrane, leading to the release of calcium ions, which activate actin and myosin in the muscle. The role of calcium in human physiology is well beyond the scope of this paper. However, it is important to note that the structural integrity of the skeletal system will be sacrificed if needed to maintain proper plasma calcium concentrations [41]. In our study, serum calcium levels did not decrease across the training, contrary to other studies in this population [21]. This was a positive finding. It may be that the additional calories from the supplements supported the overall health, as this population has been shown to be under-fueled during training [42]. It could also be that the physical training changes implemented by the Army since 2008 to decrease MSI and stress fractures specifically contributed to this finding.

Bone formations as assessed with P1NP levels were in the upper range and increased slightly during IET (80.8–90.9; normal range is 30–126 μg/mL). Bone resorption as assessed with CTX was high compared to expected rates for males in this age range (696.6–711.4, normal range is 170–600 ng/mL) [41]. Both P1NP and CTX were relatively high at week one compared to previously reported values in the age range of our sample [43]. P1NP was higher on average (84 ng/mL) than the 75th percentile of Australian males aged 20–29 (78 ng/mL), and CTX was 37 ug/mL higher than the 75th percentile [43]. It is possible that these pre-supplementation values were both on the higher end of the expected ranges due to the fact that blood collection occurred on the third day of training and, thus, was not a true pre-physical activity baseline. This could be one explanation for the lack of statistically significant changes in the bone formation and resorption across IET. Although our bone formation values were similar, our reported CTX values were much higher at the baseline in comparison to a recent report in military training on males in the UK [44]. The high level of bone resorption already seen by the third day of training may be due to the abrupt introduction to military training encountered during the first days of IET [42] and supports the early appearance of stress injuries in this population [1,21]. The Army is currently testing extending IET training for infantry soldiers from 14 weeks to 22 weeks and spreading out training events. This may allow more recovery time to reduce MSI, as well as adding more time to train soldier skills. Our levels of both bone formation and resorption were substantially lower than those found in a similar population of males and females in noncombat arms IET at a different location [21]. This may be due to the aforementioned Army training changes, variations in the training population, or variations in blood assessment methods.

In relation to our second aim, we found that age, fat mass, and ethnicity together were significantly related to bone formation, accounting for 44% of the variability of baseline bone formation. For every one-unit increase in age, there was a decrease of 4.39 units of bone formation when controlling for other factors. For every one-unit increase in fat mass, there was a corresponding 3.06 unit decrease in bone formation. Thus, older, heavier soldiers were more at-risk. Importantly, 32% of bone resorption was accounted for by run performance, fat mass, age, and ethnicity, with age being the primary predictor. For every year increase in age, there was a decrease in bone resorption by 26.63 units when controlling for the other factors. These findings suggest that, as soldiers age, their bone metabolism will naturally decrease. The finding that age alone was significant when controlling for all other factors suggests that age may be the most important factor of the factors that we examined for explaining the variable levels of bone resorption between IET soldiers. It is also important to note that the age range for these IET soldiers was narrow, and they were young (22 ± 3.5 years) compared to the experienced soldiers. These soldiers will deploy to active duty units; most will be dealing with increased load carriage weights and physical activity. Methods to balance bone formation and resorption will be important for keeping these soldiers healthy and uninjured to maintain force readiness. We also assessed testosterone levels in this analysis and found that testosterone levels were not significant indicators of bone metabolism marker levels.

Overall, the supplementations with CHO or WP as provided had no effects on the calcium, vitamin D, or markers of bone metabolism. There could be several reasons for this: (1) the dosage was inadequate, (2) the training stimulus was not adequate or of the wrong type, (3) the overall nutritional intake was low enough that any additional calories was beneficial, or (4) the supplements provided have no important impact on bone metabolism in untrained athletes. Prior research indicates WP supplementation improves bone metabolism [36,37]; however, most of this work has been done with female or older populations. Previous works have also indicated that WP supplementations can assist with nutritional deficiencies and improve muscular responses during military training [45,46]. Results from the assessment of dietary intake and metabolic load during another portion of this same study [42] found that IET soldiers were under-fueled throughout training due to soldier choices, time to consume calories, fatigue, and other reasons. The Army is currently working to address many of these factors. The lack of adequate calories may have offset any benefits of the supplementations. WP supplementation studies are also most frequently done on trained athletes; studies in untrained individuals may not show the same results [47,48]. WP supplementations may not provide the same benefits to those with the lower fitness levels often found in Army recruits. Our previous research did find an overall reduction in MSI in soldiers supplementing with WP or CHO compared to a non-supplemented group [33]. However, this may simply be due to the additional calories consumed.

There were several limitations to this study. We did not directly relate biomarker results to the dietary intakes of individual IET soldiers, nor were their hormonal panels obtained. Initial blood samples were collected on day three of training and, thus, did not represent a true pretraining baseline. Matching specific soldier intakes for biomarker results may have provided a better understanding of the metabolism. Fitness assessments were conducted by the unit drill sergeants and not the research team. However, the drill sergeants are highly trained and administer these assessments on a regular basis as a part of all IET soldier assessments. We chose the Army Physical Fitness Test to assess fitness, as it was already being conducted as a part of IET and did not require an addition to the already very tight training schedule. The unit cadre administered the supplements rather than a member of the research team. However, a member of the research team checked weekly with the unit cadre to record the supplements consumed and any supplements missed. Participants consumed the supplements by platoon rather than individually randomized. This was done to reduce any opportunity for mix-ups in the blinded supplement packages or sharing of the supplements. Platoons are housed on different floors and train and eat together. This also relieved some of the burden on the drill sergeants’ time. There were two different platoons consuming each supplement type to reduce any influence of platoon training on the results.

## 5. Conclusions

This study found male IET soldiers are mildly to moderately deficient in vitamin D and slightly deficient in calcium throughout training. A biomarker analysis suggests higher levels of bone resorption compared to bone formation may put IET soldiers at risk for MSI, especially stress injuries. Age, fitness, fat mass, and ethnicity were important predictors of bone metabolism and may be important to include as part of the screening in injury-prevention programs. Finally, a supplementation with WP or CHO as provided did not impact bone metabolism in the IET soldiers studied.

## Figures and Tables

**Table 1:** Week 1 characteristics by ethnicity.

Variable	Caucasian (N = 36)	Other (N = 54)	Combined (N = 90)	*p*-Value
**Age (years)**				0.52
Mean (SD)	21.806 (3.54)	22.30 (3.50)	22.10 (3.51)	
Range	18.00–35.00	18.00–32.00	18.00–35.000	
**Height (cm)**				0.002
Mean (SD)	176.97 (7.27)	171.97 (7.44)	173.97 (7.74)	
Range	160.30–191.90	157.50–189.50	157.500–191.900	
**Weight (kg)**				0.25
Mean (SD)	78.85 (13.90)	75.28 (14.41)	76.71 (14.24)	
Range	57.00–117.40	50.30–107.30	50.30–117.40	
**FFM (kg)**				0.29
Mean (SD)	61.64 (9.36)	59.45 (9.72)	60.33 (9.59)	
Range	49.04–84.81	43.08–87.94	43.08–87.94	
**FM (kg)**				0.35
Mean (SD)	17.21 (6.73)	15.82 (6.92)	16.38 (6.84)	
Range	6.43–32.59	1.57–35.14	1.57–35.14	
**P1NP (ng/mL)**				0.72
N	15	32	47	
Mean (SD)	86.63 (40.09)	81.82 (45.15)	84.17 (42.31)	
Range	33.68–184.38	36.52–200.20	33.68–200.20	
**CTX (ng/L)**				0.09
N	10	21	31	
Mean (SD)	788.43 (282.30)	654.68 (307.66)	713.62 (301.76)	
Range	377.12–1557.95	235.82–1589.58	235.82–1589.58	
**Vitamin D (ng/mL)**				0.04
N	16	33	49	
Mean (SD)	24.50 (9.25)	19.42 (5.82)	21.90 (8.01)	
Range	14.10–45.67	7.71–27.56	7.71–45.67	
**Calcium (mg/dL)**				0.68
N	16	24	40	
Mean (SD)	8.37 (0.49)	8.42 (0.43)	8.40 (0.45)	
Range	7.50–9.30	7.60–9.40	7.50–9.40	

SD, standard deviation; P1NP, procollagen type 1N propeptide; CTX, C-terminal crosslinking telopeptide of type 1 collagen, kg, kilogram; FM, fat mass; and FFM, fat-free mass; N, number in group.

**Table 2:** Coefficients for each regression model. These coefficients are the values related to the significant models for predicting bone formations and resorptions and can be used to predict these outcomes. Coefficients are presented as coefficient and 95% CI (lower, upper).

Parameter	P1NP	CTX
(Intercept)	87.77 ***	767.51 ***
	(74.68, 100.85)	(664.41, 870.60)
Age (years)	−4.39 **	−26.63 *
	(−7.28, −1.50)	(−49.35, -3.90)
FM (kg)	−3.06 ***	−11.54
	(−4.63, −1.48)	(−23.37, 0.29)
Ethnicity (CC/Other)	−15.54	−110.29
	(−34.29, 3.21)	(−248.26, 27.67)
Run Time (s)		−0.93
		(−2.12, 0.26)
N	42	59
R^2^	0.48	0.32

CI, confidence interval; P1NP, procollagen type 1N propeptide; CTX, C-terminal crosslinking telopeptide of type 1 collagen; kg, kilogram; CC, Caucasian; and s, seconds. *** *p* < 0.001, ** *p* < 0.01, and * *p* < 0.05.

**Table 3:** Summary of effect sizes for supplement groups across each physiological biomarker.

Variable	Mean Diff (SD)	Effect Size (95% CI)	Category
**P1NP**			
WP	3.577 (23.639)	0.084 (−0.168 to 0.337)	Negligible
CHO	5.857 (23.343)	0.135 (−0.098 to 0.368)	Negligible
**CTX**			
WP	−10.513 (262.353)	−0.040 (−0.458 to 0.378)	Negligible
CHO	14.752 (236.728)	0.043 (−0.236 to 0.322)	Negligible
**Ratio**			
WP	0.006 (0.060)	0.088 (-0.347 to 0.523)	Negligible
CHO	0.016 (0.044)	0.342 (-0.082 to 0.766)	Small
**Calcium**			
WP	−0.138 (0.511)	−0.333 (−0.981 to 0.316)	Small
CHO	−0.067 (0.440)	−0.142 (−0.53 to 0.245)	Negligible
**Vitamin D**			
WP	3.255 (6.142)	0.380 (0.024 to 0.735)	Small
CHO	4.722 (3.814)	0.965 (0.559 to 1.371)	Large

WP, whey protein; CHO, carbohydrate; P1NP (bone formation), procollagen type 1N propeptide; CTX (bone resorption), C-terminal crosslinking telopeptide of type 1 collagen; and ratio (formation/resorption ratio). Cohen’s D effect sizes; SD, standard deviation; and CI, confidence interval.

## Abbreviations

IET	Initial Entry Training
P1NP	Procollagen type 1N propeptide
CTX	C-terminal crosslinking telopeptide of type 1 collagen
FM	Fat Mass
FFM	Fat Free Mass
WP	Whey Protein
CHO	Carbohydrate
ACSM	American College of Sports Medicine
APFT	Army Physical Fitness Test
